# Clinical Impact of Preoperative Obesity on Living-Donor Kidney Transplant Recipients in Japan: A Multicenter Experience

**DOI:** 10.3390/jcm15031238

**Published:** 2026-02-04

**Authors:** Ryohei Yamamoto, Mitsuru Saito, Ryuichiro Sagehashi, Tomohiko Matsuura, Shingo Hatakeyama, Hayato Nishida, Kengo Furihata, Chika Kajiwara, Mizuki Mori, Yu Aoyama, Ayato Ito, Shinya Maita, Reiichi Murakami, Hirofumi Tomita, Hisao Saitoh, Norihiko Tsuchiya, Chikara Ohyama, Wataru Obara, Tomonori Habuchi

**Affiliations:** 1 Department of Urology, Akita University Graduate School of Medicine, Akita 010-8543, Japan; yama815@med.akita-u.ac.jp (R.Y.); thabuchi@gmail.com (T.H.); 2 Department of Urology, Iwate Medical University, Shiwa 028-3695, Japan; 3 Department of Urology, Hirosaki University Graduate School of Medicine, Hirosaki 035-8562, Japan; 4 Department of Urology, Yamagata University Faculty of Medicine, Yamagata 990-9585, Japan; hnishida331@yahoo.co.jp (H.N.);; 5 Department of Urology, Iwate Prefectural Isawa Hospital, Oshu 023-0864, Japan; 6 Department of Cardiology and Nephrology, Hirosaki University Graduate School of Medicine, Aomori 036-8652, Japan; 7 Department of Urology, Oyokyo Kidney Research Institute, Hirosaki 036-8243, Japan

**Keywords:** living kidney donor transplant, preoperative obesity, kidney transplant outcomes

## Abstract

**Background:** Obesity is increasingly prevalent among kidney transplant candidates; however, its impact on graft outcomes in Asian populations is not well characterized. We evaluated the association between preoperative obesity and living-donor kidney transplantation outcomes in Japan. **Methods:** We analyzed 623 living-donor kidney transplants performed from 1998 to 2021 at six centers in northern Japan. Recipients were categorized by body mass index (BMI) at transplant, and multivariable Cox regression was employed for assessing graft outcomes. **Results:** Obesity (BMI, ≥30 kg/m^2^; *n* = 27 [4.3%]) was the strongest graft failure predictor (hazard ratio, 4.62) compared with normal-weight recipients. Moreover, overweight status (BMI, 25–29.9 kg/m^2^), acute rejection, and older donor age were independent risk factors. Despite similar rejection rates across the BMI groups, recipients with obesity exhibited persistently impaired kidney function from 1-week posttransplant to the 5-year follow-up. Patient survival was comparable across BMI groups; however, underweight status (BMI < 18.5 kg/m^2^) was associated with higher mortality. **Conclusions:** Preoperative obesity and overweight status were significant risk factors for graft failure in Japanese living-donor kidney transplant recipients. Meanwhile, the mortality rate was significantly higher in the patients with underweight status at transplant. Pre-transplant weight optimization and shared decision-making with candidates warrant consideration.

## 1. Introduction

The global prevalence of obesity continues to rise, with approximately 38% and 14% of individuals worldwide classified as overweight (BMI > 25 kg/m^2^) and obese (BMI > 30 kg/m^2^), respectively, according to World Health Organization definitions [1]. This trend has led to an increase in obesity-related comorbidities, including cardiovascular disease, diabetes, and hypertension [2], and consequently, the number of kidney transplant candidates with obesity is also growing [3,4,5]. Kidney transplantation remains the optimal treatment for end-stage renal disease, offering superior survival and quality of life compared with long-term dialysis [6,7,8]. As transplant candidates with obesity become more common, understanding the impact of preoperative obesity on posttransplant outcomes is increasingly important.

Obesity is associated with increased surgical complications and may negatively affect both patient and graft survival [9,10,11,12]. Despite these recognized risks, there is no international consensus on BMI thresholds for kidney transplant eligibility. A global survey found that although 63% of transplant centers have established BMI cutoffs, these vary widely, and candidate selection remains largely at the discretion of individual centers [13]. This lack of standardization underscores the need for more definitive evidence.

The impact of obesity on transplant outcomes may differ across populations. Although obesity is increasing in Japan, its prevalence remains lower than in Western countries, and data specific to Asian populations are limited. We aimed to evaluate the association between preoperative BMI and outcomes following living-donor kidney transplantation using a multicenter registry from the Michinoku Renal Transplant Network (MRTN) in northern Japan. The primary outcomes were death-censored graft survival and patient survival.

## 2. Materials and Methods

### 2.1. Study Design and Participants

This retrospective cohort study used data from the Michinoku Renal Transplant Network (MRTN), a regional registry comprising six transplant centers in the Tohoku region of Japan: Akita University Hospital, Hirosaki University Hospital, Oyokyo Kidney Research Institute, Iwate Medical University Hospital, Iwate Prefectural Isawa Hospital, and Yamagata University Hospital. The study included living-donor kidney transplants performed between 1998 and 2021.

From 643 recipients registered in the database, we excluded pediatric patients (< 18 years), recipients of deceased-donor grafts, those not maintained on tacrolimus-based immunosuppression, and cases with significant missing data. Only recipients undergoing primary kidney transplantation were included. The final cohort comprised 623 adult living-donor kidney transplant recipients.

The study protocol complied with the Declaration of Helsinki. The Ethics Committee of Akita University Graduate School of Medicine approved the study (approval number: 2546, approved 11 December 2019), and institutional approval was obtained at each participating site.

### 2.2. Clinical Definitions and Protocols

Demographic and clinical data for donors and recipients were extracted from the MRTN registry. Collected variables included age, sex, duration of dialysis before transplantation, history of diabetes, ABO-blood type compatibility, and human leukocyte antigen (HLA) mismatches. Renal function was assessed using the estimated glomerular filtration rate (eGFR), calculated with the specific equation developed by the Japanese Society of Nephrology for the Japanese population [14].

The primary exposure variable was pretransplant BMI. Recipients were categorized into four groups according to WHO classifications: underweight (BMI < 18.5 kg/m^2^), normal weight (BMI 18.5–24.9 kg/m^2^), overweight (BMI 25–29.9 kg/m^2^), and obese (BMI ≥ 30 kg/m^2^).

### 2.3. Outcomes and Follow-Up

The primary endpoints were death-censored graft survival and patient survival. Graft failure was defined as the return to permanent dialysis or need for retransplantation. Conventionally, delayed graft function (DGF) is defined as the requirement for dialysis within the first week after surgery. However, because this specific data point was not consistently recorded in the registry, we used the eGFR at 1-week post-transplantation as a surrogate marker for early graft recovery [15]. Post-transplant cardiovascular disease was defined as a composite endpoint including myocardial infarction, stroke, or hospitalization for congestive heart failure.

### 2.4. Immunosuppression and Complication Management

The standard maintenance immunosuppression regimen consisted of a calcineurin inhibitor (tacrolimus), an antimetabolite (mycophenolate mofetil), and corticosteroids. Induction therapy typically included basiliximab administered intraoperatively and on postoperative day 4. For cases involving ABO incompatibility or preformed donor-specific antibodies (DSA), desensitization was performed using rituximab, plasmapheresis, or splenectomy according to institutional protocols. Everolimus was used in selected cases at the discretion of the attending physician.

Acute rejection was defined as any rejection episode requiring therapeutic intervention (e.g., steroid pulse therapy) during follow-up. Although rejection is required to be based on the biopsy, biopsy indications and its treatment protocols varied across centers over the 23-year study period. To avoid underestimating rejection incidence by excluding empirically treated cases without biopsy, we adopted this clinical definition.

Cytomegalovirus (CMV) infection was defined as symptomatic antigenemia requiring antiviral treatment (valganciclovir or ganciclovir). BK polyomavirus (BKPyV)-associated nephropathy was diagnosed based on allograft biopsy findings.

### 2.5. Statistical Analysis

Statistical analyses were performed using IBM SPSS Statistics (version 28; IBM Corp., Armonk, NY, USA) and EZR (Saitama Medical Center, Jichi Medical University, Saitama, Japan), a graphical user interface for R. We used categorical BMI variables based on WHO definitions to facilitate comparison with existing literature.

Continuous variables were compared using the Mann–Whitney U test, and categorical variables were compared using the chi-square test. Graft and patient survival were estimated using the Kaplan–Meier method, and survival differences were compared using the log-rank test.

Multivariable Cox proportional hazards regression was performed to identify independent risk factors for graft failure. Covariates were selected based on statistical associations and clinical relevance. Variables with *p* < 0.10 in univariable analysis were included. Key prognostic factors (recipient age, diabetes, and ABO incompatibility) were also included in the model regardless of univariable *p*-values to adjust for potential confounding. The proportional hazards assumption was tested using Schoenfeld residuals. Statistical significance was set at *p* < 0.05 (two-sided).

### 2.6. Use of Artificial Intelligence

During the preparation of this work, the authors used Gemini 3.0 (Google LLC, Mountain View, CA, USA) for English language editing and proofreading. The authors reviewed and edited the content as needed and take full responsibility for the content of the publication.

## 3. Results

### 3.1. Study Population

In this study, 623 adult living-donor kidney transplant recipients were stratified into the following four groups according to preoperative BMI: underweight (BMI, <18.5 kg/m^2^; *n* = 77 [12.4%]), normal weight (BMI, 18.5–24.9 kg/m^2^; *n* = 406 [65.2%]), overweight (BMI, 25–29.9 kg/m^2^; *n* = 113 [18.1%]), and obese (BMI, ≥30 kg/m^2^; *n* = 27 [4.3%]).

### 3.2. Baseline Recipient and Donor Characteristics

The baseline characteristics of recipients and donors are presented in Table 1. Comparing the recipient groups with obesity and normal weight revealed that diabetes prevalence was significantly higher in the group with obesity (40% vs. 17%, *p* = 0.008). Recipients with obesity demonstrated a significantly shorter mean follow-up duration (55 ± 34 months) than those with normal weight (106 ± 68 months) (*p* < 0.001). No significant differences were observed between the two groups in terms of mean age (45 ± 12 vs. 47 ± 13 years; *p* = 0.223), sex distribution, or other baseline factors. Preoperative donor-specific antibody (DSA) data were available for 523 recipients. The prevalence of preformed DSA was similar between the obesity (7.7%) and normal-weight (8.6%) groups (*p* = 1.00; see Supplementary Table S2).

Donors for recipients with obesity exhibited a significantly higher mean BMI than donors for recipients with normal weight (25.7 ± 4.2 vs. 23.7 ± 3.4 kg/m^2^; *p* = 0.012). The group with obesity demonstrated a significantly lower proportion of unrelated donors than the group with normal weight (22% vs. 44%; *p* = 0.042).

### 3.3. Posttransplant Renal Function

**Figure 1:**
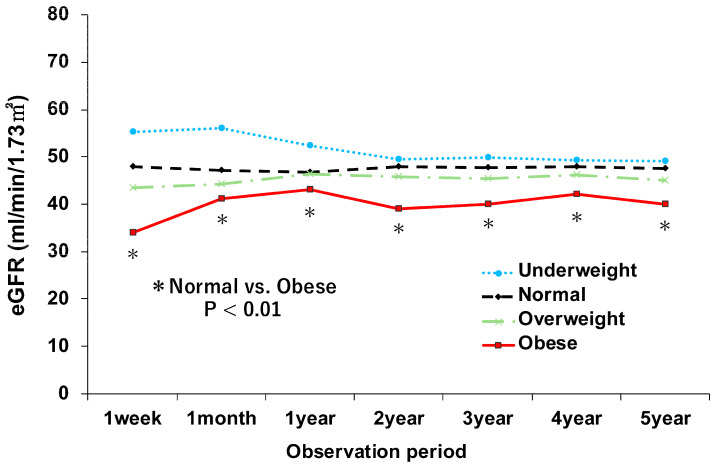
Impact of preoperative body mass index on posttransplant renal function. The asterisk (*) indicates a statistically significant difference (*p* < 0.01) between the groups with normal weight and obesity. Abbreviations: eGFR, estimated glomerular filtration rate.

The postoperative course of renal function, as measured by eGFR, is illustrated in Figure 1. This figure plots the mean eGFR changes over a 5-year follow-up period. The most prominent finding was that the group with obesity consistently demonstrated the lowest mean eGFR throughout the observation period. Although our registry does not provide formal data on DGF, the mean eGFR at 1-week posttransplant was used as a surrogate marker for early graft function. At this critical timepoint, the mean eGFR of the group with obesity was markedly lower at 34 mL/min/1.73 m^2^, whereas that of the group with normal weight was 48 mL/min/1.73 m^2^, suggesting substantially impaired early graft recovery. This disparity was statistically significant (*p* < 0.01); notably, this functional impairment was not transient but persistent, indicating that the eGFR of the group with obesity remained significantly lower than that of the group with normal weight at all measured time points from 1 week to 5 years posttransplant (*p* < 0.01).

### 3.4. Graft and Patient Survival

**Figure 2:**
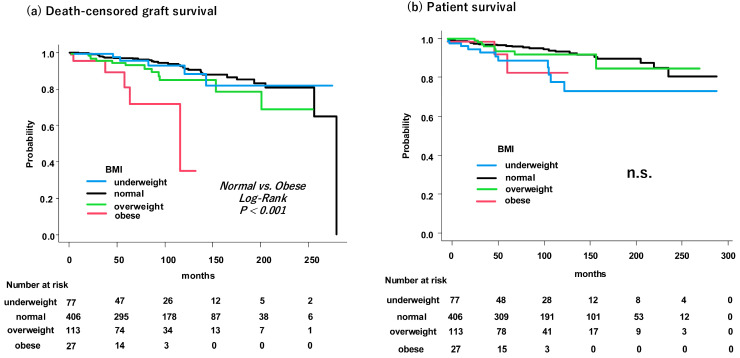
Kaplan–Meier analysis of graft and patient survival by preoperative body mass index. Kaplan—Meier curves for (**a**) death-censored graft survival and (**b**) patient survival, stratified by preoperative BMI. The group with obesity exhibits a significantly lower graft survival than the group with normal weight (log-rank *p* < 0.001). Both groups demonstrate no significant difference in terms of patient survival. Abbreviations: BMI, body mass index; n.s., not significant.

To assess survival outcomes, Kaplan–Meier analysis was performed (Figure 2). The group with obesity showed significantly lower death-censored graft survival than the group with normal weight (log-rank *p* < 0.001). The survival curve for the group with obesity revealed a more rapid decline than that for the other BMI groups. The shorter median follow-up period observed in the group with obesity indicated earlier graft failure events. In contrast, the four BMI groups demonstrated no significant differences in patient survival. In a sensitivity analysis excluding graft losses within the first 3 months, 5-year death-censored graft survival remained significantly lower in the obesity group (78.7%) than in the normal-weight group (96.6%; *p* < 0.001; Supplementary Figure S1). We also performed Cox proportional hazards analysis for patient survival. In the multivariable model, neither overweight nor obesity was associated with an increased risk of patient mortality compared to normal weight. Interestingly, however, underweight status (BMI < 18.5 kg/m^2^) was identified as a significant independent predictor of poorer patient survival (HR, 4.12; 95% CI, 1.824–7.306; *p* < 0.001). Detailed results are presented in Supplementary Table S1.

### 3.5. Posttransplant Complications

The incidence of major posttransplant complications did not differ significantly between the obesity and normal-weight groups (Table 2): acute rejection (33% vs. 39%; *p* = 0.684), CMV infection (7% vs. 9%; *p* = 0.827), BKPyV-associated nephropathy (4% vs. 7%; *p* = 0.710), and cardiovascular disease (7% vs. 13%; *p* = 0.556).

### 3.6. Independent Risk Factors for Graft Failure

A multivariable Cox proportional hazards model identified independent risk factors for death-censored graft failure (Table 3). After adjusting for confounding factors, higher BMI was identified as a significant predictor of graft loss.

Recipients with overweight (BMI, 25–29.9 kg/m^2^) demonstrated a significantly higher risk of graft failure than those with normal weight (hazard ratio [HR], 2.374; 95% confidence interval [CI], 1.248–4.514; *p* = 0.008). Recipients with obesity (BMI, ≥30 kg/m^2^) showed a substantially higher risk of graft failure (HR, 4.624; 95% CI, 2.404–7.704; *p* < 0.001).

Other independent risk factors for graft failure encompassed the presence of acute rejection (HR, 2.872; 95% CI, 1.634–5.050; *p* = 0.008) and donor age ≥ 60 years (HR, 2.213; 95% CI, 1.227–3.994; *p* = 0.008).

## 4. Discussion

The results of this study reveal that preoperative obesity strongly predicts graft failure among living-donor kidney transplant recipients, with more than a four-fold higher risk than those with normal body weight. This increased risk was observed despite comparable acute rejection rates between the groups. The consistently lower graft function observed in patients with obesity throughout the first week to 5 years posttransplant represents the most crucial observation.

We used eGFR at 1-week posttransplant as a surrogate for early graft function. Although this does not strictly correspond to DGF defined by dialysis requirement, early postoperative eGFR changes are strongly associated with long-term graft survival [16], supporting its clinical relevance as an early prognostic marker.

This early dysfunction likely results from both surgical and metabolic factors. Obesity is associated with greater technical difficulty and prolonged ischemia times [17,18]. The mismatch between a normal-sized graft and a recipient with obesity induces compensatory hyperfiltration [19], and the metabolic condition of insulin resistance and chronic inflammation further exacerbates graft injury [20]. These factors impair early graft recovery and may promote progressive fibrosis even without overt rejection [21,22].

The higher diabetes prevalence in the group with obesity (40% vs. 17%) makes this more complex. Diabetes alone harms the kidney, and the damage can worsen when combined with obesity. However, in our multivariable analysis, obesity remained a significant risk factor even after accounting for diabetes, suggesting that obesity poses independent risks beyond its association with diabetes.

Our findings differ from previous Western studies. Sureshkumar et al. reported that graft failure risk was elevated only in recipients with severe obesity (BMI > 35 kg/m^2^) in a mate-kidney analysis of the US registry [9]. In contrast, we found that BMI ≥ 30 kg/m^2^ was a significant risk factor in the Japanese population and that risk was elevated even in the overweight category (25–29.9 kg/m^2^). This discrepancy may reflect ethnic differences in body composition. Asian populations accumulate greater visceral fat at lower BMI levels compared with Caucasians [23], and visceral fat accumulation is associated with metabolic risk factors even in Japanese individuals with normal weight [24]. In our cohort, the mean BMI was 22.5 ± 3.9 kg/m^2^; thus, a BMI of ≥30 kg/m^2^ represents approximately +2 standard deviations from the mean. This suggests that Japanese patients with BMI of ≥30 kg/m^2^ constitute a metabolically distinct population in Japan, and that lower BMI thresholds (e.g., <30 kg/m^2^ or <25 kg/m^2^) may be appropriate for risk stratification in Asian kidney transplant candidates.

Patient survival was comparable across BMI groups; however, this finding requires careful interpretation. The shorter follow-up in the obesity group reflects both informative censoring due to earlier graft loss and the increasing number of kidney transplant candidates with obesity in recent years. Since prognosis after graft failure depends on dialysis-related factors rather than pre-transplant BMI, the competing risk of graft loss may mask differences in patient survival. Of note, our analysis revealed that underweight status (BMI < 18.5 kg/m^2^), rather than obesity, was associated with higher mortality. This is consistent with the obesity paradox observed in patients with chronic kidney disease, where low BMI may reflect malnutrition-inflammation complex syndrome [25].

These findings have practical implications for candidate selection in kidney transplantation. Rather than refusing transplantation based solely on BMI, shared decision-making is warranted. The substantial risk of graft failure should be discussed transparently with candidates. For those with BMI ≥ 30 kg/m^2^, structured weight loss programs and optimization of metabolic parameters before transplantation should be considered. Bariatric surgery may be an option for selected patients at centers with such programs [26].

The management of transplant candidates with obesity is evolving. GLP-1 receptor agonists and SGLT2 inhibitors, which were not widely used during our study period, offer potential strategies for perioperative weight management and metabolic control. Whether these agents can mitigate obesity-related risks for graft failure warrants investigation.

Our study has several limitations inherent to its retrospective registry design. First, the small number of patients with obesity (*n* = 27) limited statistical precision and precluded meaningful subgroup analyses. In addition, given that our findings regarding BMI thresholds differ from Western data, the generalizability of this single-nation study is limited. Larger cohort studies are necessary to validate these ethnic-specific risks. Second, detailed surgical and immunological profiles were limited by the registry design. Specifically, we could not account for donor–recipient size mismatch (e.g., sex and weight disparity), a critical determinant of nephron mass adequacy relative to recipient metabolic demand. Additionally, data on ischemia times and immediate post-transplant dialysis requirement were unavailable; given that obesity can technically complicate surgery and prolong ischemia, the lack of these variables limits our assessment of early graft injury. Furthermore, the specific causes of graft failure and a comprehensive profile of postoperative complications were not recorded, preventing a distinction between surgically driven and immunologically driven graft loss in this high-risk population. Third, the lower proportion of unrelated (spousal) donors in the obesity group (22% vs. 44%) suggests selection bias; physicians may have preferred biologically related donors for high-risk candidates, and thus our cohort may represent a fitter subset of patients with obesity. Finally, metabolic parameters such as cystatin C and HbA1c were not available. Future prospective studies incorporating these parameters are needed to validate our findings.

In summary, obesity significantly increases the risk of graft loss in living-donor kidney transplant recipients. This risk should be incorporated into shared decision-making regarding pre-transplant weight management.

## 5. Conclusions

In this multicenter cohort in Japan, both preoperative obesity (BMI ≥ 30 kg/m^2^) and overweight status (BMI 25–29.9 kg/m^2^) were identified as significant independent risk factors for early graft loss after kidney transplantation. Recipients with obesity demonstrated persistently impaired graft function throughout the 5-year follow-up, despite comparable acute rejection rates. In contrast, underweight status, rather than obesity, was associated with higher patient mortality, consistent with the obesity paradox. These findings support the need for Asian-specific BMI thresholds in transplant candidacy evaluation. Given the limited sample size (*n* = 27 [4.3%] with obesity) in this study, these results should be considered hypothesis-generating. Pre-transplant weight optimization and shared decision-making with candidates are warranted, although whether weight control improves graft outcomes requires prospective validation.

## Figures and Tables

**Table 1:** Baseline Clinical and Demographic Characteristics of Kidney Transplant Recipients and Donors, Stratified by Recipient BMI.

	BMI < 18.5	18.5 ≤ BMI < 25	25 ≤ BMI < 30	30 ≤ BMI	*p*-Value
	Underweight	Normal	Overweight	Obese	Normal vs. Obese
	N = 77	N = 406	N = 113	N = 27	
Recipient					
Age (years), mean ± SD	39 ± 14	47 ± 13	48 ± 13	45 ± 12	0.223
Sex (male), *n* (%)	27 (35)	268 (66)	85 (75)	18 (66)	0.944
Preemptive, *n* (%)	23 (29)	92 (22)	33 (29)	7 (25)	0.643
Dialysis period (month), median (IQR)	10 (4–49)	18 (5–52)	16 (3–31)	15 (0–40)	0.410
HLA mismatch, median (IQR)	3 (2–4)	3 (2–4)	3 (2–5)	4 (2–5)	0.370
ABO-incompatible, *n* (%)	22 (29)	102 (25)	25 (22)	6 (35)	0.823
BMI (kg/m^2^), mean ± SD	17.2 ± 1.1	21.6 ± 1.8	26.7 ± 1.3	33.5 ± 3.6	<0.001
Diabetes, *n* (%)	6 (8)	69 (17)	33 (29)	11 (40)	0.008
Donor					
Age (years), mean ± SD	54 ± 11	58 ± 10	57 ± 11	61 ± 10	0.308
Sex (male), *n* (%)	42 (55)	154 (38)	42 (37)	15 (56)	0.101
Unrelated donor, *n* (%)	27 (35)	177 (44)	48 (42)	6 (22)	0.042
BMI, mean ± SD	23.1 ± 3.7	23.7 ± 3.4	24.1 ± 3.8	25.7 ± 4.2	0.012
eGFR (mL/min/1.73 m^2^), mean ± SD	83.5 ± 16.2	84.3 ± 16.5	83.7 ± 15.7	81.9 ± 13.1	0.432

Data are presented as mean ± standard deviation (SD), number (%), or median [interquartile range (IQR)] for variables with skewed distributions (duration of dialysis and number of HLA mismatches). *p*-values were calculated using Student’s *t*-test or Mann–Whitney U test for continuous variables, and the Chi-square test or Fisher’s exact test for categorical variables, as appropriate. Abbreviations: BMI, body mass index; HLA, human leukocyte antigen; eGFR, estimated glomerular filtration rate.

**Table 2:** Incidence of Major Post-Transplant Complications, Stratified by Recipient BMI.

	BMI < 18.5	18.5 ≤ BMI < 25	25 ≤ BMI < 30	30 ≤ BMI	*p*-Value
	Underweight	Normal	Overweight	Obese	Normal vs. Obese
	N = 77	N = 406	N = 113	N = 27	
Acute rejection, *n* (%)	23 (30)	160 (39)	44 (33)	9 (33)	0.684
CMV infection, *n* (%)	13 (17)	35 (9)	12 (14)	2 (7)	0.827
BKPyVAN, *n* (%)	5 (6)	30 (7)	0	1 (4)	0.710
Cardiovascular Disease, *n* (%)	6 (8)	53 (13)	13 (11)	2 (7)	0.556

Abbreviations: BMI, body mass index; CMV, cytomegalovirus; BKPyVAN, BK polyoma virus associated nephropathy.

**Table 3:** Univariate and Multivariable Cox Proportional Hazards Analysis of Risk Factors for Death-Censored Graft Failure.

	Univariate Analysis				Multivariable Analysis	
	HR	95% CI	*p*-Value	HR	95% CI	*p*-Value
Recipient Factors						
Age (≥60 vs. <60 years)	0.782	0.352–1.739	0.547	0.641	0.293–1.403	0.541
Gender (male vs. female)	1.383	0.784–2.440	0.263			
BMI category (kg/m^2^)						
BMI < 18.5	0.982	0.381–2.527	0.97			
18.5 ≤ BMI < 25	Ref.			Ref.		
25 ≤ BMI < 30	2.007	1.067–3.773	0.031	2.374	1.248–4.514	0.008
30 ≤ BMI	3.778	2.189–5.251	<0.001	4.624	2.404–7.704	<0.001
Diabetes (yes vs. no)	1.324	0.661–2.653	0.428	1.625	0.761–3.221	0.358
ABO-incompatible (yes vs. no)	0.784	0.383–1.606	0.506			
Acute rejection (yes vs. no)	2.509	1.437–4.380	0.001	2.872	1.634–5.050	0.008
CMV infection (yes vs. no)	1.566	0.767–3.200	0.218			
Donor Factors						
Age (≥60 vs. <60 years)	2.132	1.243–3.656	0.006	2.213	1.227–3.994	0.008
Gender (male vs. female)	1.056	0.619–1.801	0.841			
Unrelated	0.545	0.292–1.017	0.056	0.741	0.393–1.203	0.266
BMI (≥25 vs. <25 kg/m^2^)	1.397	0.824–2.369	0.215			
eGFR (≥70 vs. <70 mL/min/1.73 m^2^)	1.515	0.865–2.652	0.146			

Abbreviations: HR, Hazard Ratio; CI, Confidence Interval; BMI, Body Mass Index; eGFR, estimated glomerular filtration rate; CMV, cytomegalovirus; Ref., Reference category.

## Data Availability

The datasets generated and/or analyzed during this study are not publicly available owing to patient privacy protection but are available from the corresponding author on reasonable request.

## Supplementary Materials

The following supporting information can be downloaded at: https://www.mdpi.com/article/10.3390/jcm15031238/s1, Table S1. Univariate and Multivariable Cox Proportional Hazards Analysis of Risk Factors for Patient survival; Table S2: Prevalence of Preformed Donor-Specific Antibodies (DSA), Stratified by Recipient Body mass index (BMI); Figure S1. Sensitivity analysis of death-censored graft survival excluding early graft losses.

## Abbreviations

The following abbreviations are used in this manuscript:

BMI	Body mass index
BKPyV	BK polyoma virus
CMV	Cytomegalovirus
DGF	Delayed graft function
eGFR	estimated glomerular filtration rate
HR	hazard ratio
MRTN	Michinoku Renal Transplant Network
WHO	World Health Organization
