# Supplementary material for: Clinical Impact of Preoperative Obesity on Living-Donor Kidney Transplant Recipients in Japan: A Multicenter Experience
Source: J Clin Med. 2026 Feb 4;15(3):1238. doi: 10.3390/jcm15031238 (PMC12898629; doi:10.3390/jcm15031238)
Supplement: Supplementary file 1 [file jcm-15-01238-s001.zip › jcm-4071994-supplementary-Table S2.pdf]

Supplementary Table S2. Prevalence of Preformed Donor-Specific Antibodies (DSA), Stratified by Recipient Body mass index (BMI).

	**BMI ＜18.5 Underweight n = 74**	**18.5≦BMI＜25 Normal n = 326**	**25≦BMI＜30 Overweight n = 97**	**30≦BMI Obese n = 26**	**P-value Normal vs. Obese**
Preformed DSA, *n* (%)	7 (9.5)	28 (8.6)	11 (11.3)	2（7.7）	1.00

Data for preformed donor-specific antibodies were available for 523 out of 623 recipients. *p*-values were calculated using Fisher's exact test.
