# Supplementary material for: Clinical Impact of Preoperative Obesity on Living-Donor Kidney Transplant Recipients in Japan: A Multicenter Experience
Source: J Clin Med. 2026 Feb 4;15(3):1238. doi: 10.3390/jcm15031238 (PMC12898629; doi:10.3390/jcm15031238)
Supplement: Supplementary file 1 [file jcm-15-01238-s001.zip › jcm-4071994-supplementary.pdf]

Supplementary Table S1. Univariate and Multivariable Cox Proportional Hazards Analysis of Risk Factors for Patient survival

	Univariable analysis			Multivariable analysis		
	HR	95% CI	*p*-value	HR	95% CI	*p*-value
**Recipient Factors**						
Age (≥ 60 vs. < 60)	2.311	1.157-4.615	0.018	3.411	1.622-7.170	0.001
Gender （male vs. female)	2.108	1.006-4.420	0.048	2.700	1.261-5.865	0.011
BMI category						
BMI＜18.5	2.582	1.231-5.416	0.012	4.120	1.824-7.306	< 0.001
18.5 ≤ BMI＜25	Ref.			Ref.		
25 ≤ BMI ＜ 30	0.859	0.361-2.045	0.732			
30 ≤ BMI	1.670	0.468-6.289	0.355	2.325	0.713-9.169	0.215
Diabetes (yes vs. no)	1.324	0.661 - 2.653	0.428			
**Donor Factors**						
Age (≥ 60 vs. < 60)	1.005	0.986-1.024	0.611			
Gender (male vs. female)	0.804	0.528-1.223	0.307			
BMI (≥25 vs. < 25)	1.397	0.824-2.369	0.215			

Abbreviations: HR, Hazard Ratio; CI, Confidence Interval; BMI, Body Mass Index; Ref., Reference category

Supplementary Figure S1. Sensitivity analysis of death-censored graft survival excluding early graft losses.

Kaplan–Meier estimates for death-censored graft survival stratified by recipient BMI categories, excluding graft losses occurring within the first 3 months post-transplantation. Despite excluding early failures to account for potential immortal time bias, the obesity group (BMI ≥ 30 kg/m²) demonstrated significantly inferior graft survival compared to the normal- weight group (log-rank $p < 0.001$)

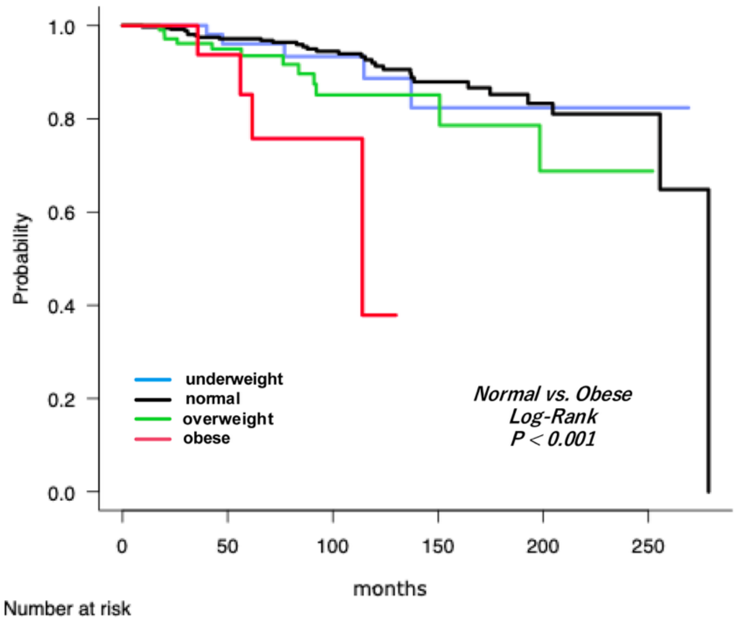

Number at risk

underweight	77	47	26	12	5	2
normal	404	295	178	87	38	6
overweight	111	74	34	13	7	1
obese	26	14	3	0	0	0
